# Shifts in the climate space of temperate cyprinid fishes due to climate change are coupled with altered body sizes and growth rates

**DOI:** 10.1111/gcb.13230

**Published:** 2016-03-07

**Authors:** Ana Ruiz‐Navarro, Phillipa K. Gillingham, J. Robert Britton

**Affiliations:** ^1^ Centre for EcologyEnvironment and SustainabilityDepartment of Life and Environmental SciencesBournemouth UniversityPooleDorsetBH12 5BBUK

**Keywords:** climate change, climate envelope, emissions, lowland rivers, somatic growth

## Abstract

Predictions of species responses to climate change often focus on distribution shifts, although responses can also include shifts in body sizes and population demographics. Here, shifts in the distributional ranges (‘climate space’), body sizes (as maximum theoretical body sizes, *L*∞) and growth rates (as rate at which *L*∞ is reached, *K*) were predicted for five fishes of the Cyprinidae family in a temperate region over eight climate change projections. Great Britain was the model area, and the model species were *Rutilus rutilus*,* Leuciscus leuciscus*,* Squalius cephalus*,* Gobio gobio* and *Abramis brama*. Ensemble models predicted that the species' climate spaces would shift in all modelled projections, with the most drastic changes occurring under high emissions; all range centroids shifted in a north‐westerly direction. Predicted climate space expanded for *R. rutilus* and *A. brama*, contracted for *S. cephalus*, and for *L. leuciscus* and *G. gobio*, expanded under low‐emission scenarios but contracted under high emissions, suggesting the presence of some climate‐distribution thresholds. For *R. rutilus*,* A. brama*,* S. cephalus* and *G. gobio*, shifts in their climate space were coupled with predicted shifts to significantly smaller maximum body sizes and/or faster growth rates, aligning strongly to aspects of temperature‐body size theory. These predicted shifts in *L*∞ and *K* had considerable consequences for size‐at‐age per species, suggesting substantial alterations in population age structures and abundances. Thus, when predicting climate change outcomes for species, outputs that couple shifts in climate space with altered body sizes and growth rates provide considerable insights into the population and community consequences, especially for species that cannot easily track their thermal niches.

## Introduction

Climate change will be a major driver of biodiversity changes throughout this century (Sala *et al*., 2000), with evidence that species across a range of taxonomic groups are already responding to recent climatic change by shifting their ranges (Root *et al*., 2003; Hickling *et al*., 2006; Comte *et al*., 2013). These shifts may then result in substantial changes at the assemblage level (Stralberg *et al*., 2009; Comte *et al*., 2014; Markovic *et al*., 2014). Responses to climate change are not limited to range shifts, however. Rising temperatures have substantial consequences for the biology and ecology of species, such as in their phenology, including timing of reproduction (e.g. Thackeray *et al*., 2010; Pankhurst & Munday, 2011; Krabbenhoft *et al*., 2014), reproductive traits (Crozier & Hutchings, 2014), age structure (Jeppesen *et al*., 2012), body sizes (Daufresne *et al*., 2009) and growth rates (Morrongiello *et al*., 2014). Whilst there remains considerable uncertainty in the extent of the biological and ecological consequences that will result from warming (Parmesan *et al*., 2013; Wenger *et al*., 2013), understanding the full extent of responses of species remains crucial for initiating appropriate management strategies (Graham & Harrod, 2009; Dawson *et al*., 2011).

Evidence suggests that, to date, the response of species to climate change is relatively consistent across ecosystems, regions and taxa (Daufresne *et al*., 2009; Thomas, 2010; Poloczanska *et al*., 2013). Common responses are distribution shifts polewards and/or to increased altitudes (Chen *et al*., 2011; Bebber *et al*., 2013; Comte & Grenouillet, 2013), as these facilitate species tracking their climate niches (Crimmins *et al*., 2011). For this to be successful requires the rate of distribution change to match the pace of isotherm shifts, that is the climate change velocity (Isaak & Rieman, 2013). Evidence suggests, however, that this is rarely the case (Zhu *et al*., 2012; Corlett & Westcott, 2013). For example, movements in many plant species will need to be in excess of 1 km per year to track climate change velocities, with the majority of species unable to so this (Corlett & Westcott, 2013). In French populations of stream fishes, range shifts also generally lagged behind the pace of isotherm shifts, especially at the range centre (Comte & Grenouillet, 2013, 2015). As most studies focus on identifying the individual species that will either benefit or decline due to warming (Domisch *et al*., 2011; Rosset & Oertli, 2011), there have been relatively few attempts to identify the mechanisms underpinning the responses of species (Wenger *et al*., 2011). Where this has been completed, it has often focused on the analysis of functional traits in relation to climate change responses, particularly in plants (e.g. Soudzilovskaia *et al*., 2013). It thus remains uncertain as to how many species will respond to warming temperatures within their original ranges (Comte & Grenouillet, 2015), despite the ecological importance of these responses given the potential time lag between range change and isotherm shifts in taxa such as freshwater fishes (Comte & Grenouillet, 2013).

In this regard, fluvial fishes are strong model species for both predicting how climate change could induce range shifts and assessing how warming in the original range affects their biology and ecology, such as in their body sizes and the expression of life history traits (Britton *et al*., 2010a). Their ectothermic physiology, high trait plasticity to environmental conditions (especially temperature) and constrained distributions within river basins all provide favourable attributes for predicting responses to warming (Comte *et al*., 2014). For many species and communities, especially in lowland rivers, their population dynamics tend to be largely density‐independent and strongly influenced by climate variables (e.g. Nunn *et al*., 2007; Beardsley & Britton, 2012). To date, their reported responses to climate change tend to align with those of other taxa, with predictions of reduced ranges for cold‐water species and the converse for cool‐ and warm‐water species, especially in temperate regions (Comte *et al*., 2013). There is less certainty on the life history trait responses of fish to warming, where studies have primarily focused on marine systems (Blanchard *et al*., 2012; Heath *et al*., 2012), with predictions including increased growth, production and abundance in the middle of the species range, but reduced growth at range edges (Rijnsdorp *et al*., 2009; Neuheimer *et al*., 2011), and with populations often comprising of individuals of smaller body sizes (Baudron *et al*., 2014). This has also been detected in some freshwater communities (Daufresne & Boët, 2007), with Daufresne *et al*. (2009) suggesting reduced body size is the third universal ecological response to warming in aquatic systems following range change and seasonal shifts in life cycles. In marine fishes, the reduced body sizes result from factors including the impacts of temperature changes on ecological and metabolic rules (Sheridan & Bickford, 2011), and the interaction of shifts to earlier sexual maturation and growth rate increases due to elevated temperatures (Neuheimer & Grønkjær, 2012).

For fluvial fishes in temperate regions, the primary focus of their responses to warming has been on range shifts and associated community and functional diversity (e.g. Buisson *et al*., 2013; Comte *et al*., 2013, 2014), with less emphasis on other aspects of their population ecology. Consequently, the aim here was to predict, for a range of climate change projections in a model temperate region in northern Europe, how shifts in the distributional ranges of five model fluvial fishes were coupled to predicted shifts in their growth rates and body sizes. We predicted that in all climate change projections, predictions of range changes for the model fishes would be coupled with a shift to smaller maximum body sizes and concomitant changes in their growth rates, but with the extent of these changes being species‐specific.

## Materials and methods

### Study area and fish species

The model region was Great Britain, which has sufficient latitude and longitudinal ranges to provide marked differences in regional climates and thus differences in climate change projections. The five cyprinid fishes were selected on the basis of their presence and coexistence in fish assemblages in lowland British rivers (< 200 m altitude): roach *Rutilus rutilus*, chub *Squalius cephalus*, dace *Leuciscus leuciscus*, gudgeon *Gobio gobio* and common bream *Abramis brama*. Their distributions also stretch across Eurasia. Their maximum reported body sizes vary between species (*R. rutilus*: 500 mm, *S. cephalus*: 620 mm, *A. brama* 820 mm, *L. leuciscus* 400 mm and *G. gobio* 200 mm) (www.Fishbase.org; Kompowski, 1982; Britton, 2007), and in rivers, their population dynamics are strongly influenced by climate variables (e.g. Beardsley & Britton, 2012). Consequently, the body size and growth rate analyses used data from only fluvial populations.

### Fish distribution data

The occurrences of the fishes within Great Britain were obtained from the ‘Database for the Atlas of Freshwater Fishes’, provided by the Biological Records Centre and available at the NBN Gateway website (https://data.nbn.org.uk/Datasets/GA000174). The majority of the records ranged from 1950 to 2003 in the British National Grid spatial reference system (based on the 1936 Ordnance Survey Great Britain datum, OSGB_36) at a 10 × 10 km resolution, prior to their conversion to the World Geodetic System WGS_84 grid system. It should be noted that all 10‐km grid squares in Great Britain contain part of a river, hence use of this system. Species absences were considered to be sampled locations in Great Britain where fish species other than the model species were present in the ‘Database for the Atlas of Freshwater Fishes’.

### Fish length and growth data

Data on the body lengths and growth rates of the fishes were available from fluvial populations in England and Wales (comparable data were not available from Scotland, preventing predictions using data from higher latitudes). These data were available as length‐at‐age data of individual fishes, taken from scales that were collected during river fisheries monitoring surveys by the Environment Agency of England and Wales between 2000 and 2005. The scales had been aged on a projecting microscope, with errors minimized using the quality control procedure of Musk *et al*. (2006). There were 43 populations used for *R. rutilus* (maximum age of an individual fish: 10 years old; mean number of fish per population 90.9 ± 14.0 SE), 32 for *S. cephalus* (15 years; 63.3 ± 7.0), 20 for *A. brama* (15 years; 39.6 ± 8.4), 31 for *L. leuciscus* (8 years; 53.2 ± 6.9) and 30 for *G. gobio* (5 years; 35.8 ± 5.8). For all species, length‐at‐age data from fluvial populations were used as growth rates of species such as *R. rutilus* and *A. brama* tend to be largely density‐dependent in lentic situations (e.g. Burrough & Kennedy, 1979; Linfield, 1979, 1980).

The ageing data were used to calculate the back‐calculated length at the last annulus for each individual fish, as calculated by the scale proportional method (Francis, 1990). For each species and population, these data were used in a two‐parameter von Bertalanffy growth model of the form

(1) $$L_{t}=L\infty \left(1-\exp^{-\mathrm{kt}}\right)$$

(Eqn (1)), where *L*_t_ was the actual length of each fish at observed age t, *L*∞ was the asymptotic length (i.e. maximum theoretical body size for the population, referred to hereafter simply as maximum body size) and *K* was the growth coefficient (i.e. its annual growth rate to *L*∞, referred to hereafter as the growth rate). Model fitting was as per Britton *et al*. (2010b). Consequently, the data available for modelling the growth parameters of each population per species were their location (grid square), and their von Bertalanffy growth model parameters of their maximum theoretical body size (*L*∞) and growth rate to *L*∞ (*K*).

### Climate data

Both baseline (averages for the period 1960 to 1990) and future global projections of climate data (annual values) were obtained from the WorldClim website (http://www.worldclim.org/), version 1.4 (release 3), at a 5‐min resolution in the WGS_84 grid system. Climate projections for the years 2050 and 2070, under low‐ (rcp 2.6) and high (rcp 8.5)‐emission scenarios were obtained from two different climate prediction models: BCC‐CSM1‐1 and HadGEM2‐AO (*n *=* *9). A ‘UK outline polygon’, obtained from the OS Opensource website (https://www.ordnancesurvey.co.uk/opendatadownload/products.html), was used to clip the climatic data to the area for Great Britain. The 19 climatic variables available, derived from the monthly temperature and rainfall values, were reduced to six through analysis of their correlations, whereby variables with Pearson's correlation coefficients of above a threshold 0.70 were not duplicated in the climate data set. Consequently, the climatic variables used were as follows: annual mean temperature (°C), mean diurnal range of temperature (°C), isothermality [100 × (mean diurnal range/annual range of temperature)], mean temperature of wettest quarter (°C), mean temperature of driest quarter (°C) and annual precipitation (mm). The rationale for retaining these variables rather than their correlates was as per Araújo *et al*. (2006), as they reflect the two primary properties of the climate, energy and water that have strong abiotic and biotic influences on the distribution and ecology of freshwater fishes, although it is acknowledged that these climate variables are not the only determinants of fish distribution (Pont *et al*., 2006).

### Fish distribution‐climate modelling

Fish species distributions in Great Britain were modelled using seven algorithms available in the *biomod2* package (Thuiller *et al*., 2014) in R: (1) generalized linear models (GLM), (2) generalized additive models (GAM), (3) multivariate adaptive regression splines (MARS), (4) classification tree analysis (CTA), (5) boosted regression trees (BRT), (6) random forests (RF) and (7) artificial neural networks. In all models, the default options of *biomod2* were selected, with the exception of restricting the GAM smoothing to 4 knots to avoid overfitting the data. Models were evaluated using the area under the ROC curve (AUC) using an 80 : 20 split of training to test data and 50 evaluation repetitions. AUC values range between 0 and 1, where 1 indicates excellent performance and values lower than 0.5 indicate predictive discrimination that is no better than a random guess. As it has been considered to significantly improve robustness of predictions (Marmion *et al*., 2009), ensemble models were then created using the weighted‐mean combination of single models with AUC ≥0.7, based on their individual AUC evaluation scores.

Once the probability of presence of each species had been estimated for each geographic grid square by the ensemble models, a threshold of probability of presence was then applied to the cells. Whilst there are a number of options for selecting this threshold, including use of the threshold probability that maximizes kappa and thus minimizes prediction error based on current climate conditions (e.g. Huntley *et al*., 2008), models that have high‐probability thresholds (e.g. 0.8) tend to less good at generalizing than models of low‐probability thresholds (e.g. 0.5), although use of the latter increases the chance of prediction error (Liu *et al*., 2005). Consequently, whilst thresholds of 0.5, 0.6 and 0.8 were tested initially, 0.6 was selected for final use, based on these trade‐offs (Liu *et al*., 2005). Following the application of this threshold, the number of grid squares that were predicted to be occupied by each species was then counted for the different scenarios, and the location of the corresponding centroids was calculated. The ensemble models were then evaluated using the ROC curve (AUC), which is used extensively in species distribution modelling (SDM) (Elith *et al*., 2006), and it is generally considered the best metric for comparisons in the same geographic space (Buisson *et al*., 2008). The centroids of the simulated present ranges and the predicted future climate spaces of each species in each climate change projection were then calculated as the points about which the sum of the distances of all the grid squares in which the species was predicted to be present was zero, with the Euclidean distances from all cells to the centroid calculated and then tested for differences from the predicted centroid under the current conditions.

The main output of the distribution‐climate modelling of the fishes for each climate change projection was, therefore, the simulated extent of the spatial area of Great Britain that populations of these fishes in current climate conditions (Fig. S1), and their predicted spatial distribution under each climate change projection (Figs S2–S6). These outputs thus indicate the extent of the range change for each species (i.e. simulated current vs. predicted projection). However, given the potential time lag between range change and isotherm shifts (Comte & Grenouillet, 2013), assuming that there are even natural dispersal opportunities available (Jackson & Sax, 2009), then we interpreted these outputs as predictions of the ‘climate space’ of the species and the extent of the change from their original climate space. It is thus acknowledged that whilst the climate space of the species might alter with a changing climate, this does not necessarily imply there will be a concomitant change in the actual distribution of that species.

### Maximum body size and growth rate climate modelling

The maximum body sizes (*L*∞) and growth rates (*K*) of each population per species were modelled separately, using each as the dependent variable and their location's climatic variables as the independent variables. To maintain consistency with the distribution‐climate modelling, for each location, the same six climatic variables were used, with the rationale of their selection described above. These variables were then applied to three different algorithms: (1) GLM, (2) GAM and (3) MARS. GLM was adjusted with the ‘iteratively reweighted least squares’ method. In GAM, knots were limited to 4 and smooth terms were penalized with either of two possible regression splines: thin plate regression splines and cubic regression splines. All different possible combinations of smooth terms were tested, and, for each species, the one with minimum generalized cross‐validation score (GCV) was selected. In MARS, the threshold of 0.001 was chosen as the minimum R^2^ change to step forward, together with 30 cross‐validations. GCV was taken as an estimate of the mean square prediction error based on a leave‐one‐out cross‐validation estimation process, with lower values showing an improved fit. Again, to improve robustness of predictions (Marmion *et al*., 2009), ensemble models were created using the weighted‐mean combination of single models, based on their individual GCV scores. The goodness of fit of the ensemble models was assessed by means of root mean squared error between the original values of *K* and *L*∞ and their corresponding values predicted by the selected growth models under the original climatic conditions. Thus, the model outputs were the predicted values of *L*∞ and *K* values for each population under each climate change projection.

### Integrating outputs from distribution‐, maximum body size‐ and growth rate climate models

The outputs of the three modelling approaches were integrated via analysis of their predicted values for each of the eight climate change projections. Correspondingly, the mean (± 95% confidence limits) predicted maximum body sizes (*L*∞) and growth rates (*K*) were determined for each climate change projection per species and tested against the predicted change in the climate space for that species in that scenario (linear regression). Given that *L*∞ and *K* are closely related variables that generally explain the growth patterns of these species in Great Britain (Britton, 2007), their predicted values were then used to calculate and plot the mean lengths at age per species using Eqn (1) from the climate change projections and used to identify how their altered values affected their lengths‐at‐age. This was completed via comparisons of their predicted values from the original data, the low‐emission 2050 BCC‐CSM1‐1 scenario and the high‐emission HadGEM2‐AO 2070 scenario, thus encompassing the full range of the predicted values in relation to the climate change projections.

Where error is presented around the mean, it represents standard error (SE) unless stated otherwise. Parametric tests were only used following normality and homogeneity of variances.

## Results

### Roach *Rutilus rutilus*

**Figure 1:**
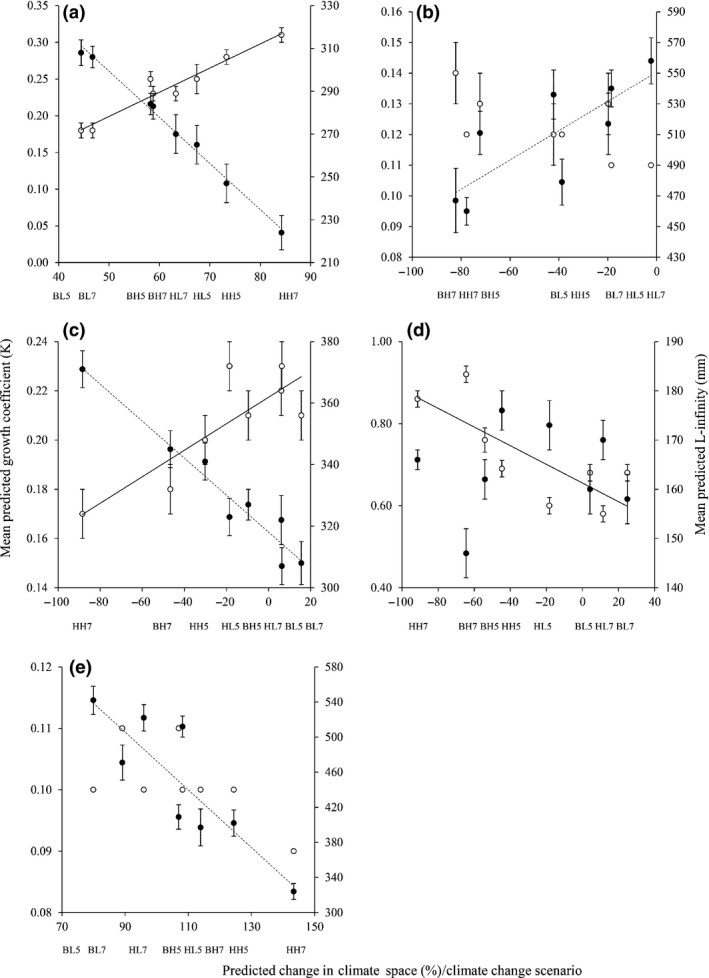
Mean (± SE) predicted growth coefficient (*K*; ○) and mean predicted maximum theoretical length (*L*∞) vs. the predicted change in climate space per climate change projection for (a) *Rutilus rutilus*, (b) *Squalius cephalus*, (c) *Leuciscus leuciscus,* (d) *Gobio gobio* and (e) *Abramis brama*. Solid line: significant relationship between change in climate space and *K*; dashed line: significant relationship between change in climate space and *L*∞.

**Figure 2:**
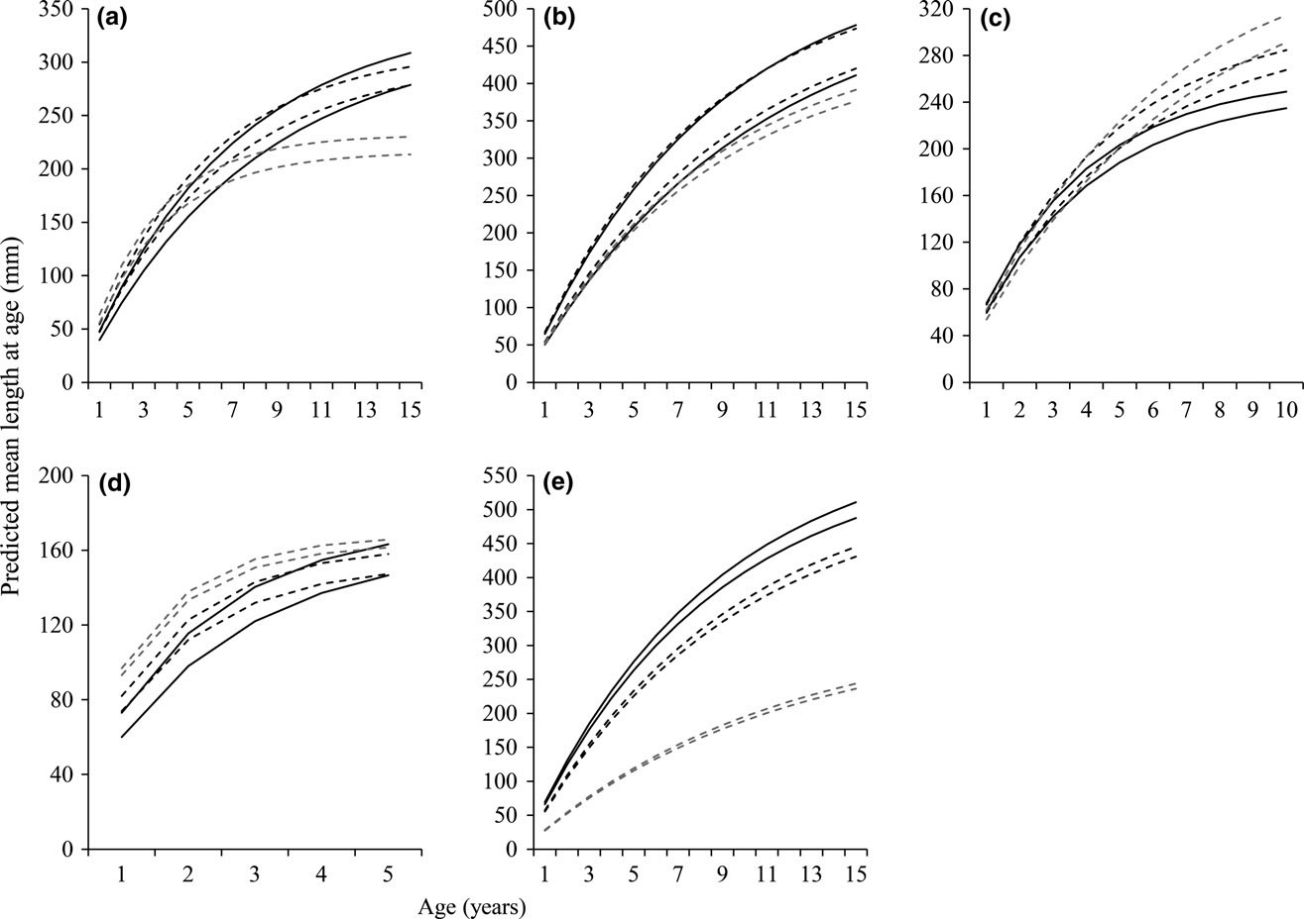
Comparison of mean length at age of the model fishes across different predicted climate scenarios: (a) *Rutilus rutilus*, (b) *Squalius cephalus*, (c) *Leuciscus leuciscus,* (d) *Gobio gobio* and (e) *Abramis brama*. Each plot shows the von Bertalanffy growth curves (95% confidence intervals) for the original data (solid lines), the low‐emission scenario of BCC‐CSM1‐1 2050 (black dashed line) and the high‐emission projection for HadGEm2‐AO (grey dashed line). Note differences in scales on the axes.

The ensemble model for *R. rutilus* had an AUC 0.95 and predicted that their climate space in Great Britain would expand in each climate change projection, with increased space with higher emissions (44 to 84%; Table 1; Fig. S2). Their direction of centroid displacement was north‐westerly, with distance varying with the modelled scenario, with larger displacements under high‐emission scenarios (Table 2). The differences between the longitude and latitude of their original and predicted centroids were significant in all projections (*t‐*tests; Table 2). These predictions of increased climate space with projections of increasing emissions were coupled with significantly reduced maximum body sizes (*L*∞) and significantly faster annual growth rates (*K*) (*L*∞: *R* ^2^ = 0.99; *F*_1,6_ = 828.41, *P *<* *0.01; *K*:* R* ^2^ = 0.94; *F*_1,6_ = 91.72, *P *<* *0.01; Fig. 1). In the low‐emission scenarios, these alterations in body size and growth rate had only minor consequences for their mean lengths at age, but in the high‐emission scenarios, they resulted in rapid growth early in life but with substantial slowing thereafter (Fig. 2).

**Table 1:** Number of grid cells (squares) occupied by the species at present (simulated data) and in the projected future scenarios, and percentage change (%) with respect to the original simulated distribution of the species. Low ES: low‐emission scenario; High ES: high‐emission scenario

			BCC‐CSM1‐1				HadGEM2‐AO			
	Original (No. squares)	Prediction (Year)	Low ES		High ES		Low ES		High ES	
			No. squares	Change	No. squares	Change	No. squares	Change	No. squares	Change
*Rutilus rutilus*	1154	2050	1667	+44.45	1826	+58.23	1932	+67.42	2000	+73.31
		2070	1693	+46.71	1832	+58.75	1884	+63.26	2126	+84.23
*Squalius cephalus*	782	2050	452	−42.20	217	−72.25	637	−18.54	479	−38.75
		2070	627	−19.82	139	−82.23	764	−2.30	174	−77.75
*Abramis brama*	807	2050	1452	+79.93	1670	+106.94	1680	+108.18	1811	+124.41
		2070	1526	+89.10	1726	113.88	1581	+95.91	1964	+143.37
*Leuciscus leuciscus*	853	2050	907	+6.33	771	−9.61	695	−18.52	596	−30.13
		2070	986	+15.59	455	−46.66	905	+6.10	99	−88.39
*Gobio gobio*	884	2050	847	−4.19	406	−54.07	722	−18.33	489	−44.68
		2070	1104	+24.89	314	−64.48	985	+11.43	76	−91.40

**Table 2:** Location (latitude and longitude, decimal degrees) of the centroids of the original distribution of the model fishes and predicted changes in projected emission scenarios (ES) (km, and bearing in arc degrees considering 0° the north and increasing values in a clockwise direction). Also, the results of the Student's *t‐*test (*t‐*statistic and significance) of their respective comparisons with original distributions are presented (Lat *t‐*test: *t‐*test of comparing latitude location; Long *t‐*test: *t‐*test of longitude location). (a) BCC‐CSM1‐1, (b) HadGEM2‐AO

Species	Original		Year	Low ES				High ES			
	Long	Lat		Distance	Lat (t)	Long(t)	Bearing	Distance	Lat (t)	Long(t)	Bearing
(a) BCC‐CSM1‐1											
*R. rutilus*	−1.497	52.777	2050	75	−10.5^a^	5.4^a^	343.8	97	−13.2^a^	8.6^a^	340.1
			2070	89	−12.4^a^	6.1^a^	344.6	101	−13.7^a^	8.6^a^	340.8
*S. cephalus*	−1.262	52.511	2050	178	−16.6^a^	12.0^a^	344.4	361	−37.5^a^	28.4^a^	343.2
			2070	175	−19.07^a^	15.7^a^	340.3	370	−38.5^a^	26.1^a^	343.1
*A. brama*	−1.185	52.600	2050	74	−9.91^a^	6.4^a^	338.4	102	−13.5^a^	10.0^a^	335.9
			2070	89	−12.13^a^	7.5^a^	339.4	112	−14.9^a^	10.8^a^	336.7
*L. leuciscus*	−1.228	52.468	2050	59	−8.2^a^	−2.8^a^	11.7	95	−12.0^a^	0.01	359.9
			2070	76	−11.25^a^	−1.4^a^	4.3	168	−16.9^a^	4.4^a^	352.6
*G. gobio*	−1.286	52.535	2050	135	−17.6^a^	2.7^a^	355.1	245	−21.8^a^	9.8^a^	347.6
			2070	108	−15.4^a^	2.5^a^	354.6	244	−19.8^a^	9.9^a^	347.2

Species	Original		Year	Low ES				High ES			
	Long	Lat		Distance	Lat (t)	Long(t)	Bearing	Distance	Lat (t)	Long(t)	Bearing
(b) HadGEM2‐AO											
*R. rutilus*	−1.497	52.777	2050	84	−10.9^a^	10.6^a^	330.5	99	−12.9^a^	11.8^a^	332.4
			2070	77	−10.0^a^	9.9^a^	329.5	121	−15.7^a^	13.9^a^	334.0
*S. cephalus*	−1.262	52.511	2050	265	−32.4^a^	23.9^a^	340.0	342	−39.0^a^	30.5^a^	341.0
			2070	168	−21.1^a^	19.6^a^	332.9	398	−38.3^a^	30.3^a^	341.0
*A. brama*	−1.185	52.600	2050	70	−8.0^a^	11.1^a^	317.6	93	−11.6^a^	12.8^a^	325.2
			2070	54	−5.4^a^	9.9^a^	308.2	117	−14.6^a^	15.1^a^	327.7
*L. leuciscus*	−1.228	52.468	2050	177	−21.9^a^	3.6^a^	354.3	219	−22.1^a^	9.7^a^	347.6
			2070	95	−13.9^a^	2.6^a^	353.0	336	−13.4^a^	4.3^a^	350.2
*G. gobio*	−1.286	52.535	2050	226	−28.7^a^	12.4^a^	345.6	291	−28.8^a^	15.3^a^	345.1
			2070	138	−19.4^a^	9.8^a^	342.1	505	−35.3^a^	28.2^a^	344.4

a *P *≤* *0.01.

### Chub *Squalius cephalus*

For the *S. cephalus* ensemble model, AUC was 0.95, with a predicted contraction of their climate space in each climate change projection (−19 to −82%; Table 1; Fig. S3). As with *R. rutilus*, the direction of centroid displacement was north‐westerly and whilst the distance of displacement varied with the modelled scenario, large displacements were predicted under high‐emission scenarios (maximum distance: 398 km; Table 2). The predicted decrease in their climate space with scenarios of increasing emissions was coupled with a predicted shift to significantly smaller maximum body sizes (*L*∞) (*R* ^2^ = 0.63; *F*_1,6_ = 10.39, *P *<* *0.02; Fig. 1). However, predictions were for little change in their annual growth rates (*K*), with no relationship with climate space change (*R* ^2^ = 0.45; *F*_1,6_ = 4.94, *P *=* *0.07 (Fig. 1). In combination, these outputs meant that there was predicted to be little change in their mean lengths at age in low‐emission scenarios, but with some reduction in their mean lengths at age in higher emissions (Fig. 2).

### Dace *Leuciscus leuciscus*

The *L. leuciscus* ensemble model had an AUC of 0.95 and predicted that their changes in climate space would vary with the projection; expansions were generally predicted under low emissions (to 16%) but with contractions under high emissions (to −88%) (Table 1; Fig. S4). Again, the direction of centroid displacement was north‐westerly, with displacement of 336 km in the high‐emission HADGEM2‐AO scenario (Table 2), with these shifts being significant from the original predicted centroids (*t‐*tests; Table 2). As their predictions of climate space decreased with increasing emissions, their mean predicted maximum body sizes (*L*∞) were predicted to significantly increase, from 307 mm in the lowest emission scenario to 371 mm in the highest emission scenario (*R* ^2^ = 0.97; *F*_1,6_ = 97.10, *P *<* *0.01; Fig. 1), the only species in which this was apparent. This was coupled with a concomitant significant decrease in their growth rate (*K*) (*R* ^2^ = 0.71; *F*_1,6_ = 14.30, *P *<* *0.01). In all cases, these predicted alterations in body size and growth rate resulted in elevated lengths at age in all climate change projections, but with this most apparent in the projections from the higher emissions, especially from the age of 5 years (Fig. 2).

### Gudgeon *Gobio gobio*

The AUC of the *G. gobio* ensemble model was 0.94, and, similar to *L. leuciscus*, the predicted changes in their climate space across the climate change projections were variable, with expansions under low emissions (to 25%) and contractions under high emissions (to −91%) (Table 1; Fig. S5). The direction of centroid displacement was north‐westerly, with larger displacement distances in high‐emission scenarios (Table 2) and significant differences between the longitude and latitude of the original and predicted centroids (*t‐*tests; Table 2). As their predicted climate space decreased with scenarios of increasing emissions, their mean predicted maximum body sizes (*L*∞) showed little variation (147 to 176 mm; Fig. 1), with the relationship between mean predicted *L*∞ and the predicted change in climate space being not significant (*R* ^2^ = 0.01; *F*_1,6_ = 0.04, *P *=* *0.84). Conversely, their growth rate (*K*) increased significantly as their climate space decreased with higher emissions (*R* ^2^ = 0.61; *F*_1,6_ = 9.52, *P *<* *0.03) (Fig. 1). These changes in *L*∞ and *K* across the scenarios resulted in their initial lengths at age being elevated in higher emissions scenarios, but with their lengths at age 4 and 5 years then being relatively similar across all projections (Fig. 2).

### Common bream *Abramis brama*

The ensemble model for *A. brama* (AUC: 0.94) predicted that their climate space would increase by between 80 and 143%, with increased predicted climate spaces in high‐emission scenarios (Table 1; Fig. S6). Predictions of centroid displacement were between 70 and 117 km in a north‐westerly direction, with significant differences in their locations to the original predicted centroids (*t‐*tests; Table 2). As their predicted climate space increased with scenarios of increasing emissions, their mean predicted maximum body sizes (*L*∞) significantly decreased, from 542 mm (predicted 80% increase in climate space) to 324 mm (predicted 143% increase in climate space) (*R* ^2^ = 0.76; *F*_1,6_ = 18.84, *P *<* *0.01; Fig. 1). In contrast, there was minimal change predicted in their growth rate (*K*), with this not significantly related to predicted changes of climate space (*R* ^2^ = 0.37; *F*_1,6_ = 3.53, *P *=* *0.11) (Fig. 1). This predicted substantial shift in maximum body sizes with higher emissions then resulted in considerable decreases in their predicted mean lengths at age (Fig. 2).

## Discussion

It was predicted that for three of the model species, *R. rutilus*,* A. brama* and *S. cephalus*, shifts in their climate space (i.e. their potential future distribution range) under the climate change projections were coupled with shifts to populations comprising of individuals of significantly smaller maximum theoretical body sizes that would grow faster, resulting in predictions of reduced lengths at age under high‐emission climate change projections. For *R. rutilus* and *A. brama*, these changes were coupled with predictions of increasing climate space with higher emissions, whereas for *S. cephalus*, predictions were for contractions in climate space, suggesting their climate optimum in Great Britain might be exceeded in future and emphasizing some species‐specificity in the climate change responses. For *G. gobio*, predicted reductions in their climate space were not coupled with predictions of significantly reduced body sizes but were to a shift to populations comprising of faster growing individuals. It was only *L. leuciscus* where predictions were for a shift to larger body sizes and slower growth under high‐emission scenarios and when their climate space was predicted to substantially contract. The reasons for their exception to the general patterns predicted for body sizes and growth rates were not clear.

These predictions under high‐emission climate change projections of shifts to smaller body and/or faster growth rates in four of the model fishes align strongly to aspects of temperature‐body size theory. For example, James’ rule suggests that in warmer environments, populations of a given species will comprise individuals of smaller body sizes (James, 1970), whilst the temperature‐size rule (TSR) suggests that for ectotherms, individual body sizes tend to decrease with increasing temperature (Atkinson, 1994). Daufresne *et al*. (2009) built on these rules, suggesting that due to the TSR, size‐at‐ages within populations should decrease with increasing temperature (‘size‐at‐age shift hypothesis’), especially at advanced ages as the TSR predicts a higher growth rate but a lower final size at higher temperatures. Our outcomes, especially for *R. rutilus*, largely corresponded with this. Moreover, Daufresne *et al*. (2009) then suggested that following this decrease in size‐at‐age and life stage, an increase in the proportion of juveniles could also be expected at the population (‘scale population age‐structure shift hypothesis’). Although this could not be tested here, the expression of life history traits in *A. brama* and *R. rutilus* populations are highly plastic, with faster growth and earlier maturity often observed in populations in disturbed conditions (Linfield, 1980; Beardsley & Britton, 2012), and those in more southerly latitudes at higher mean air temperatures (Lappalainen *et al*., 2008; Tarkan & Vilizzi, 2015). Thus, this suggests that their future populations in Great Britain are likely to be composed of smaller, shorter‐lived, fast‐growing individuals that reproduce earlier in life. Indeed, such changes in population demographics have already been observed in marine fishes of the North Sea, where the effects are from the interaction of increased growth due to temperature coupled with earlier maturity, although fishing pressure might also be acting upon this via removal of larger bodied individuals (Baudron *et al*., 2014). Daufresne *et al*. (2009) concluded by suggesting reduced body size is the third universal ecological response to warming, at least in aquatic systems, and the majority of our outputs corroborated this. However, we also revealed that these smaller body sizes would be related to individuals growing faster and, by extension, reproducing earlier in life and at smaller body sizes (Denney *et al*., 2002). Notwithstanding, in reviewing over 30 studies completed since 2000 on climate change‐induced shifts in body size, Gardner *et al*. (2011) found varied responses over a wide range of endo‐ and ectothermic species (increases, decreases and no change), suggesting considerable heterogeneity in the magnitude and direction of size responses across taxa more generally.

The predicted directional shifts in the climate spaces of the model fishes were consistent with those from other fish‐based climate change studies, with strong evidence in both freshwater and marine systems that range changes will occur in most fishes due to climate change (Jackson & Mandrak, 2002; Chu *et al*., 2005; Rahel & Olden, 2008; Jones *et al*., 2013; Elliott *et al*., 2015). Whilst the direction and magnitude of range shifts are shaped by the species‐specific sensitivity to the changes (e.g. their physiological tolerance, resilience and potential to adapt) (Graham & Harrod, 2009; Comte & Grenouillet, 2015), the general pattern over a wide range of terrestrial and aquatic taxa is a poleward and altitudinal range shift as species track their thermal niche (e.g. Chen *et al*., 2011; Melles *et al*., 2011), including plants (Corlett & Westcott, 2013) and insects (Forister *et al*., 2010). The drivers of these range changes can be complex, with Conti *et al*. (2015) suggesting that where species had expanding ranges, this was influenced more by changes in the seasonality of temperatures, whereas where ranges contract, it is due to the interaction of temperature change and alterations in precipitation patterns. Although it was unable to be tested further here, the range contraction of *S. cephalus* predicted under all climate change projections might relate to this interaction, given their natural distribution in more southern latitudes in Europe (Tedesco *et al*., 2009), where air temperatures tend to be higher than for Great Britain. For *G. gobio* and *L. leuciscus*, predictions suggested some climate thresholds might exist, given their predicted expansions of climate space under low‐emission projections of climate space but constrictions under high emissions.

For freshwater species already at their upper thermal limit, climate change is likely to increase their vulnerability to extirpation from existing areas of their range, especially where their colonization of new climatically favourable patches may not be straightforward, such as through their inability to move from catchment to catchment (Jackson & Sax, 2009). Indeed, when discussing the potential range changes of a freshwater species, it should be considered that unlike for marine fishes, distribution shifts are heavily dependent on the interaction between species’ dispersal abilities, the connectivity of the hydrographic network and the presence of physical barriers that prevent movements (Warren *et al*., 2001; Conti *et al*., 2015). Consequently, where species are unable to move easily from their existing ranges then their population resilience could be impacted, as already observed in many salmonid fishes where higher temperatures and lower flows in summer are particular concerns from climate change (e.g. Wenger *et al*., 2011; Jones *et al*., 2014). For the cyprinid species used in this study, their movement northwards to areas outside of their pre‐existing ranges (such as to areas in Scotland and Wales) is likely to be inhibited by poor hydrological connectivity and thus would be reliant on anthropogenic assistance. Whilst this has already occurred in some instances, these have tended to be unregulated releases of *R. rutilus* into their nonindigenous ranges and, as such, there is a desire to prevent further releases at the present time (Winfield *et al*., 2008, 2011). Indeed, it is the unregulated movement of fishes, such as *R. rutilus*, to areas outside of their indigenous range that potentially present a considerable threat to the biogeography and ecological integrity to many freshwater systems in northern Britain (Winfield *et al*., 2011), with this threat magnified by climate change projections (Winfield *et al*., 2008). Prevention requires strong enforcement and regulation provided by extant legislation (Hickley & Chare, 2004), as well as education schemes to inform anglers of the danger of moving fish in an unregulated manner and without risk assessment (Rahel, 2004). Consequently, whilst predictions might suggest range expansions to the north and west in future climatic conditions, the combination of low hydrological connectivity and regulatory issues associated with their biogeography might prevent this being realized. This then suggests responses of the species will be within existing ranges, where shifts to smaller body sizes, faster growth and more abundant populations might become strongly apparent.

Although these predictions suggest considerable climate change‐induced alterations in the population demographics of these fishes, it is acknowledged that climatic variables are not the only factors that will affect the growth rates and body sizes of these fishes, with the effects of other abiotic and biotic factors also being important, including other anthropogenic disturbances such as nutrient enrichment (Beardsley & Britton, 2012). Moreover, the model fishes will be within freshwater communities comprising species that are all responding to the effects of climate change, and thus, their responses will also be governed by the altered strengths of their interspecific interactions and predator–prey relationships (Johnson *et al*., 2009; Walther, 2010). Indeed, Gilman *et al*. (2010) discussed that failing to incorporate species interactions into climate change predictions limits their accuracy at the population and community level, and so some caution is necessary around our outcomes. This also suggests that the next steps in this work are to increase model complexity by incorporating biotic interactions and dispersal opportunities (Conti *et al*., 2015). Responses of fish to climate change in marine systems, particularly in relation to body size, are also affected by exploitation, with body sizes tending to decrease with fishery activities (Kuparinen & Merila, 2007). However, for the fish populations in our study, exploitation was generally limited to low levels of catch‐and‐release angling (Aprahamian *et al*., 2010), limiting its influence on population demography. In addition, given the absence of the model fishes in some areas of northern Britain then their climate niche across the whole of the model area would not have been fully described due to these natural range limits (Maitland, 2004), thus potentially impeding some aspects of the performance of the ensemble models.

In conclusion, the predictions for the model fishes were for climate space shifts under climate change projections that were species‐specific, but that for four of the fishes, these shifts were coupled with predictions of decreased body sizes and/or a shift to faster growth rates. These outputs were generally consistent with aspects of temperature‐body size theory, particularly the ‘size‐at‐age shift hypothesis’ and, most probably, the ‘scale population age‐structure shift hypothesis’ (Daufresne *et al*., 2009). They thus have important implications for the demographics of their populations in Great Britain, and given their natural distribution, then across many Eurasian regions, although it is acknowledged that life history traits of fishes are also strongly influenced by a wide range of interacting abiotic and biotic factors. In closing, we emphasize the importance of coupling the effects of climate change on species’ climate spaces with shifts in their body sizes and growth rates, as these have considerable implications for population demographics and community structure, with such climate change‐driven shifts unlikely to be limited to stream fishes.

## Supporting information


**Figure S1**. Actual vs. predicted distributions of model species under current climate conditions.

**Figure S2**. Distribution maps of *Rutilus rutilus* at present and across different predicted climate projections.

**Figure S3**. Distribution maps of *Squalius cephalus* at present and across different predicted climate projections.

**Figure S4**. Distribution maps of *Leuciscus leuciscus* at present and across different predicted climate projections.

**Figure S5**. Distribution maps of *Gobio gobio* at present and across different predicted climate projections.

**Figure S6**. Distribution maps of *Abramis brama* at present and across different predicted climate projections.
