# Supplementary figures and images for: Shifts in the climate space of temperate cyprinid fishes due to climate change are coupled with altered body sizes and growth rates
Source: Glob Chang Biol. 2016 Mar 7;22(9):3221–32. doi: 10.1111/gcb.13230 (PMC5021213; doi:10.1111/gcb.13230)

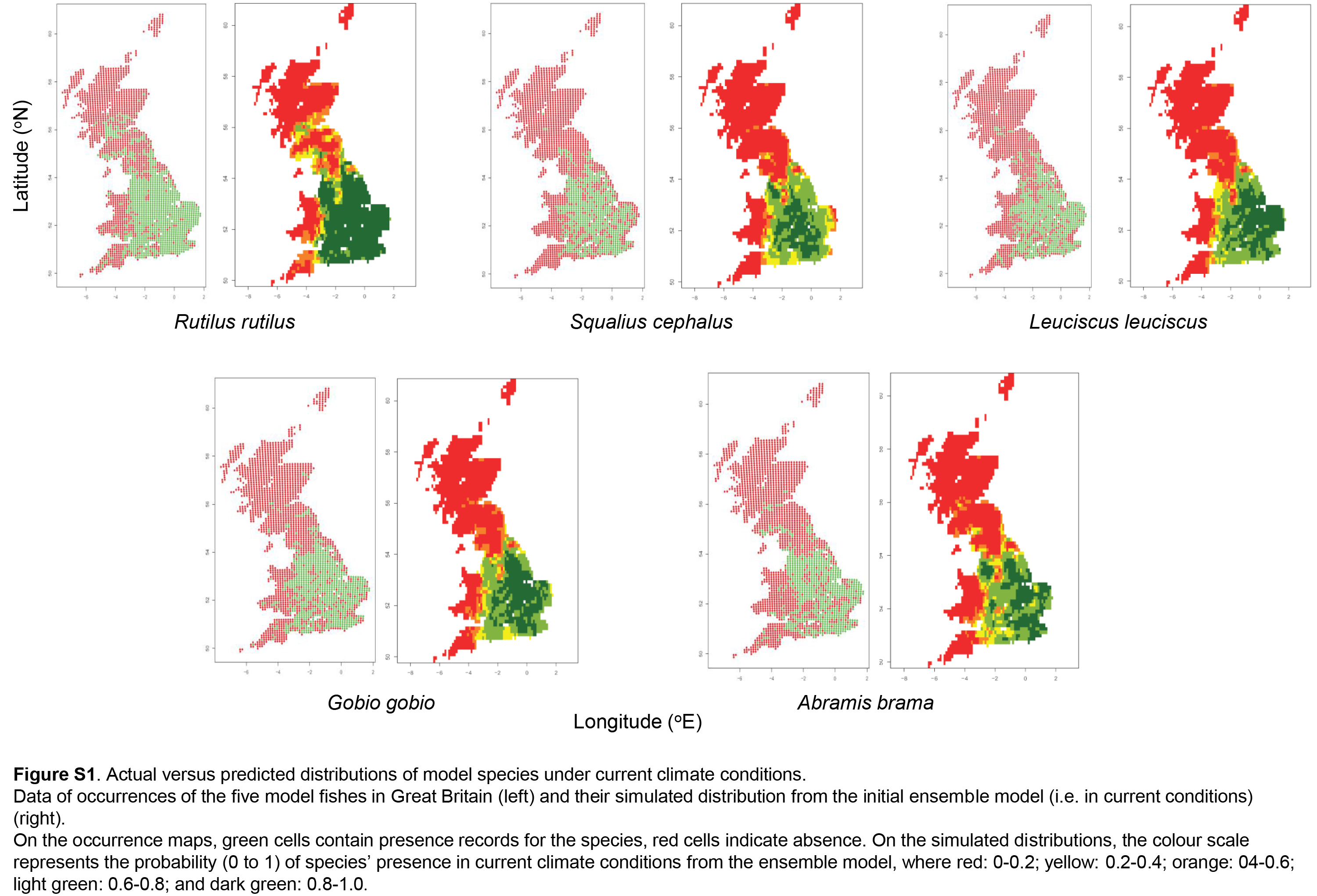

Supplement: Supplementary file 1 — Figure S1. Actual vs. predicted distributions of model species under current climate conditions. [file GCB-22-3221-s001.tif]

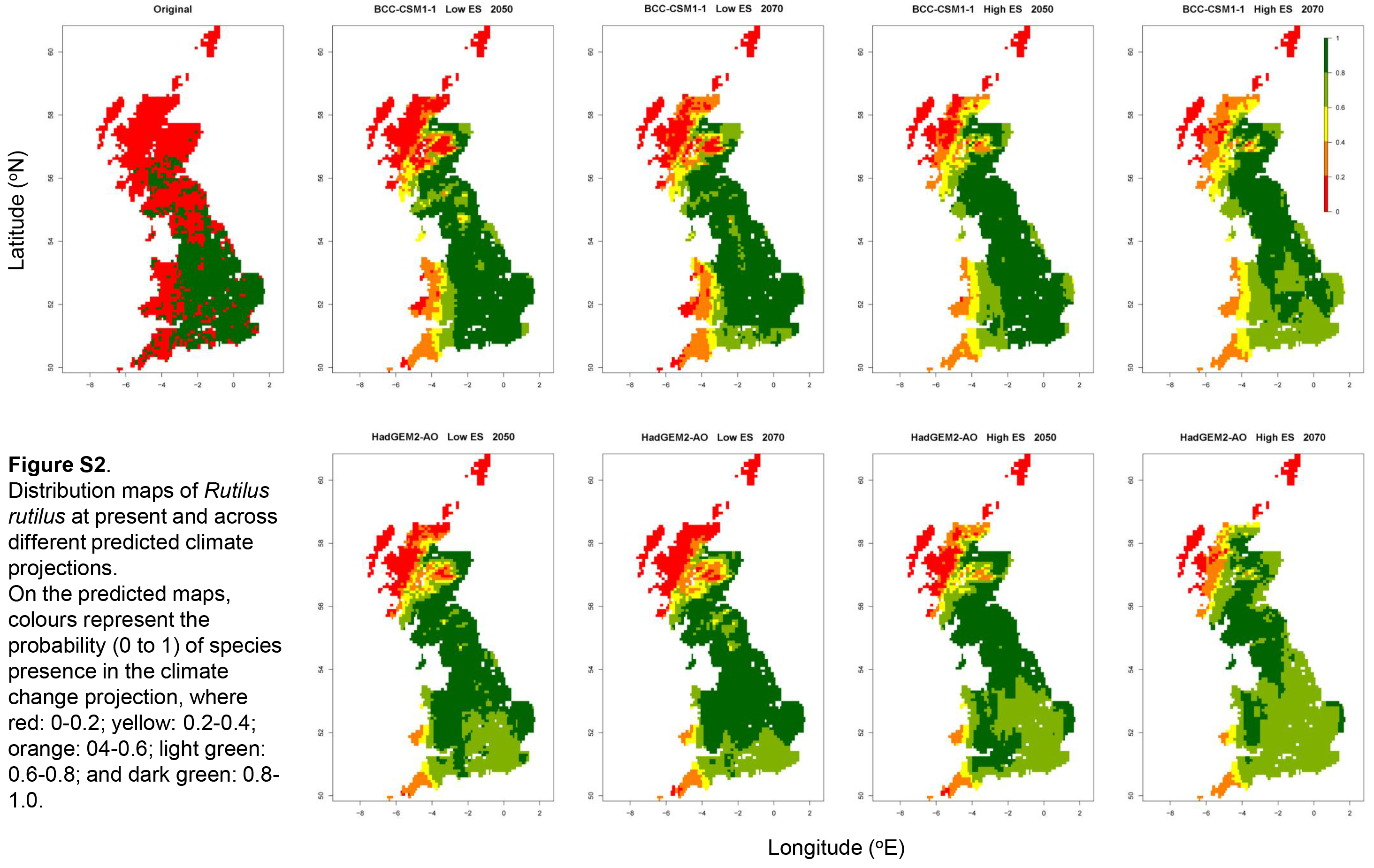

Supplement: Supplementary file 2 — Figure S2. Distribution maps of Rutilus rutilus at present and across different predicted climate projections. [file GCB-22-3221-s002.tif]

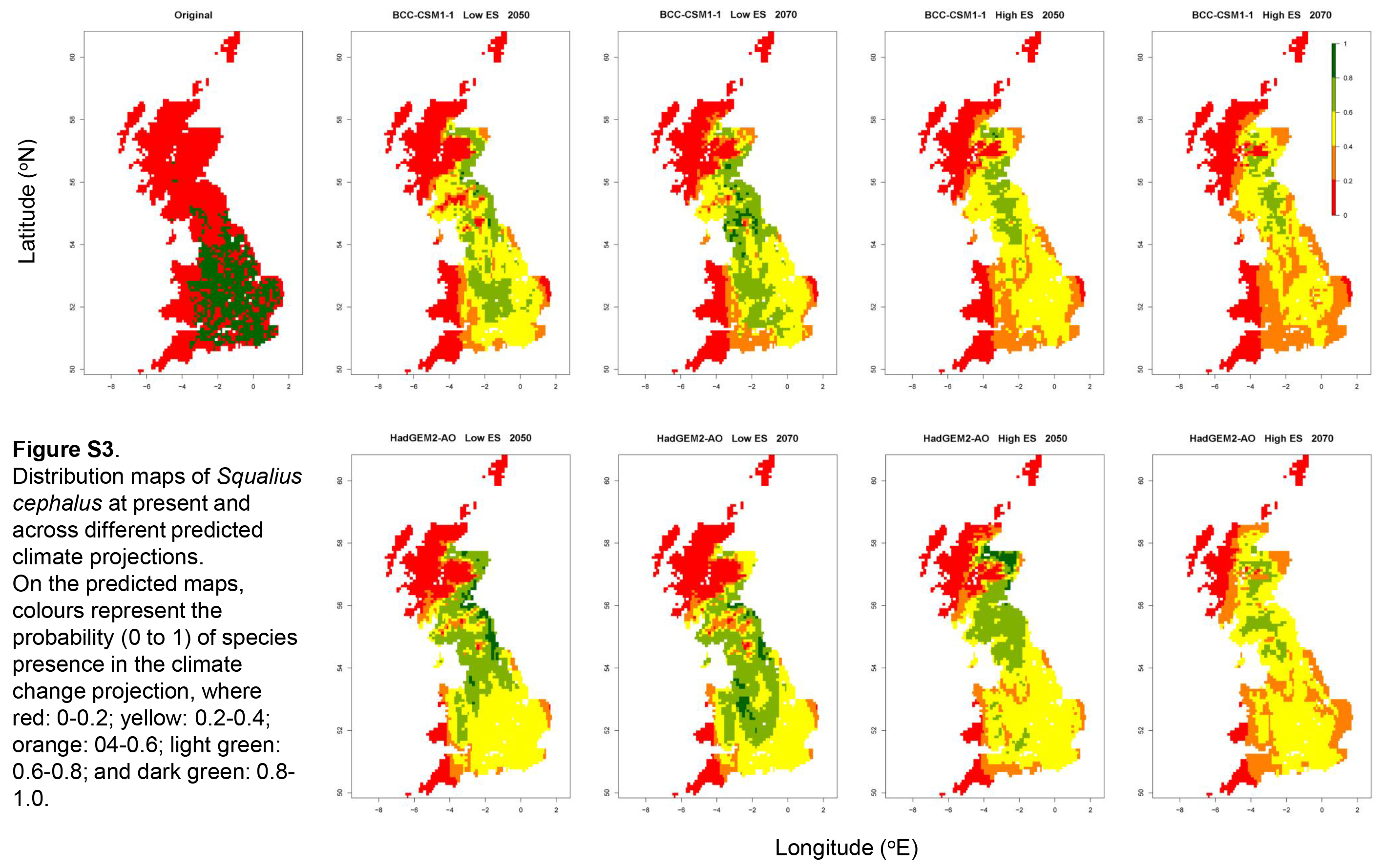

Supplement: Supplementary file 3 — Figure S3. Distribution maps of Squalius cephalus at present and across different predicted climate projections. [file GCB-22-3221-s003.tif]

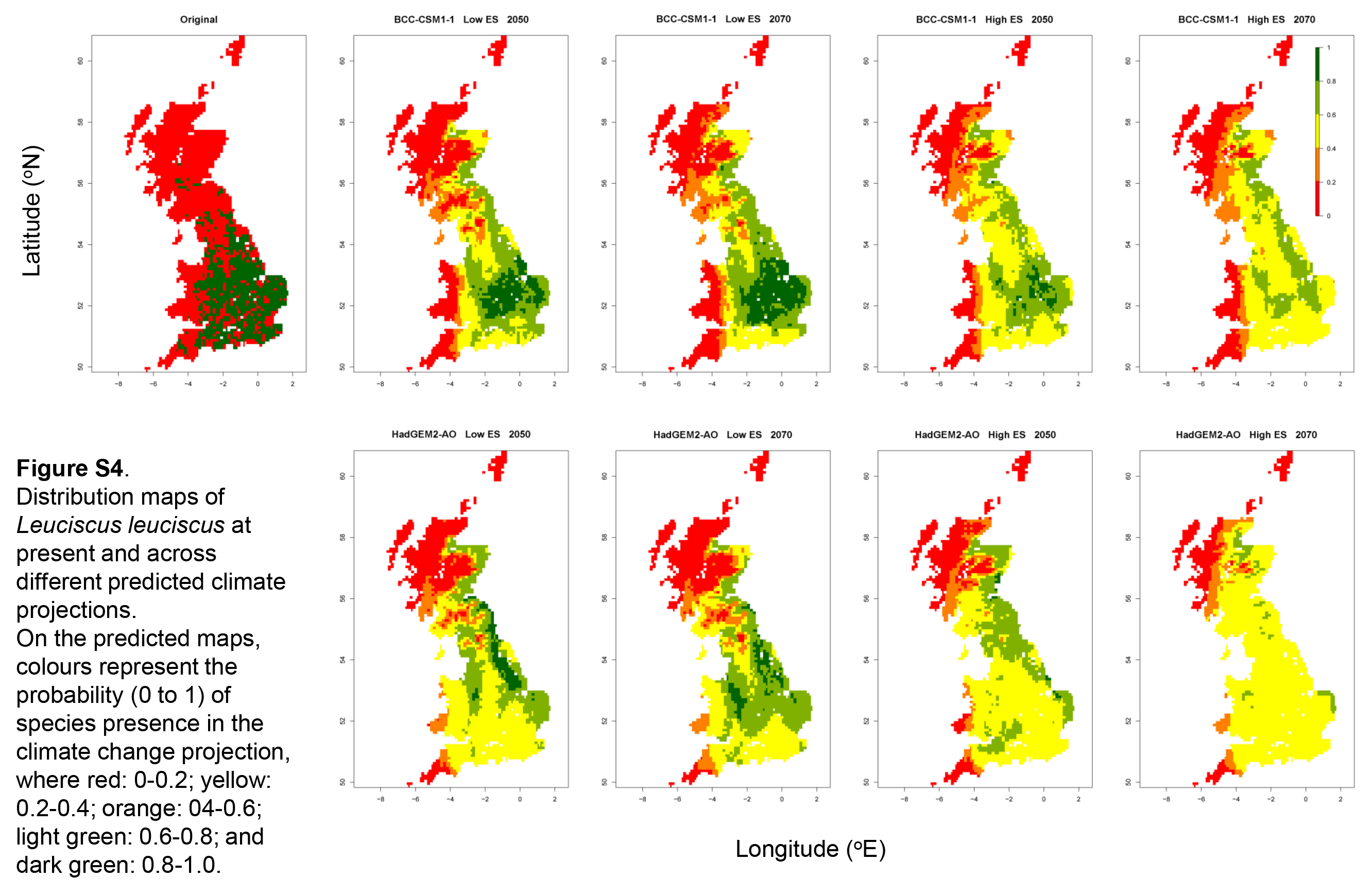

Supplement: Supplementary file 4 — Figure S4. Distribution maps of Leuciscus leuciscus at present and across different predicted climate projections. [file GCB-22-3221-s004.tif]

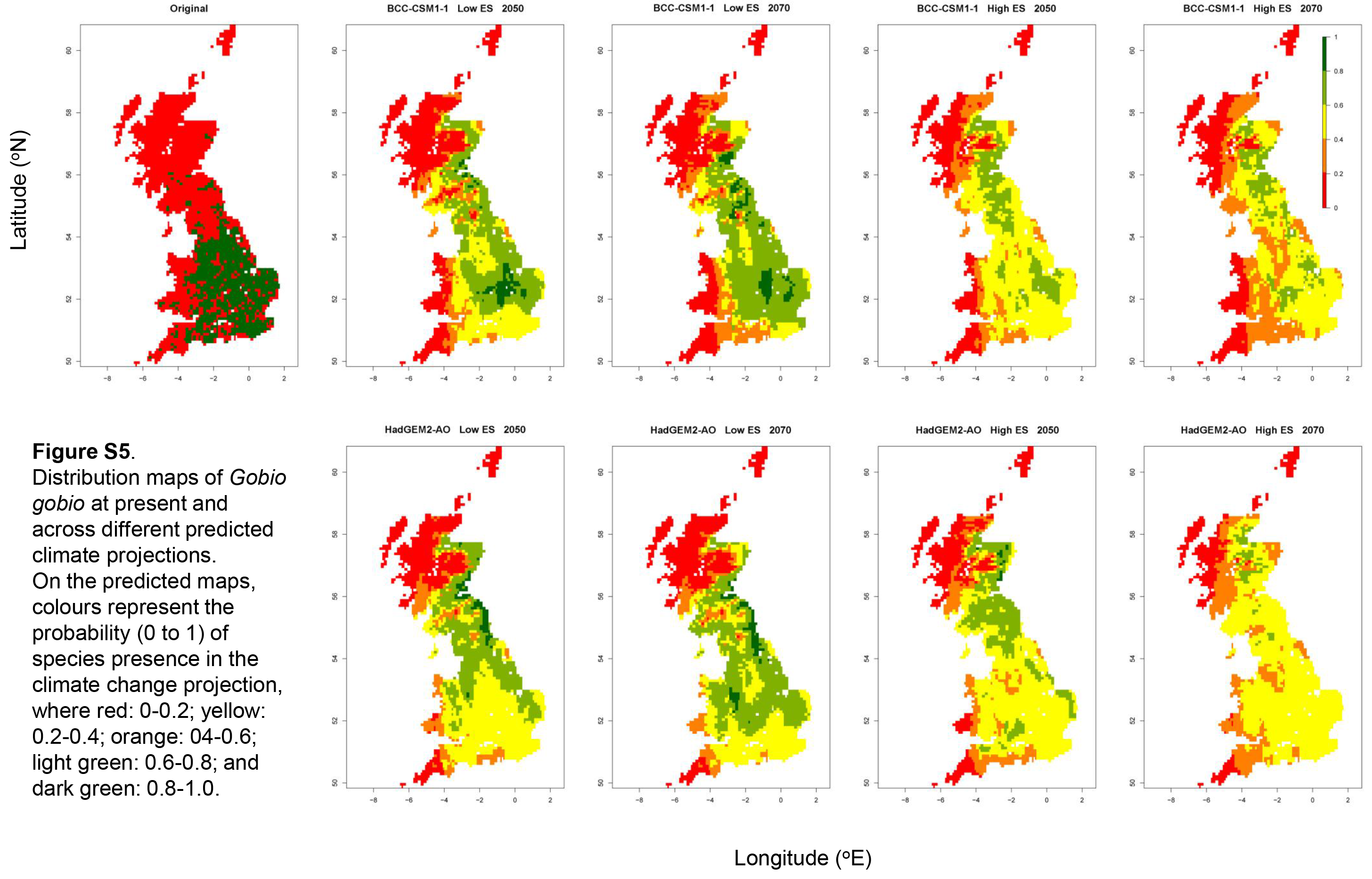

Supplement: Supplementary file 5 — Figure S5. Distribution maps of Gobio gobio at present and across different predicted climate projections. [file GCB-22-3221-s005.tif]

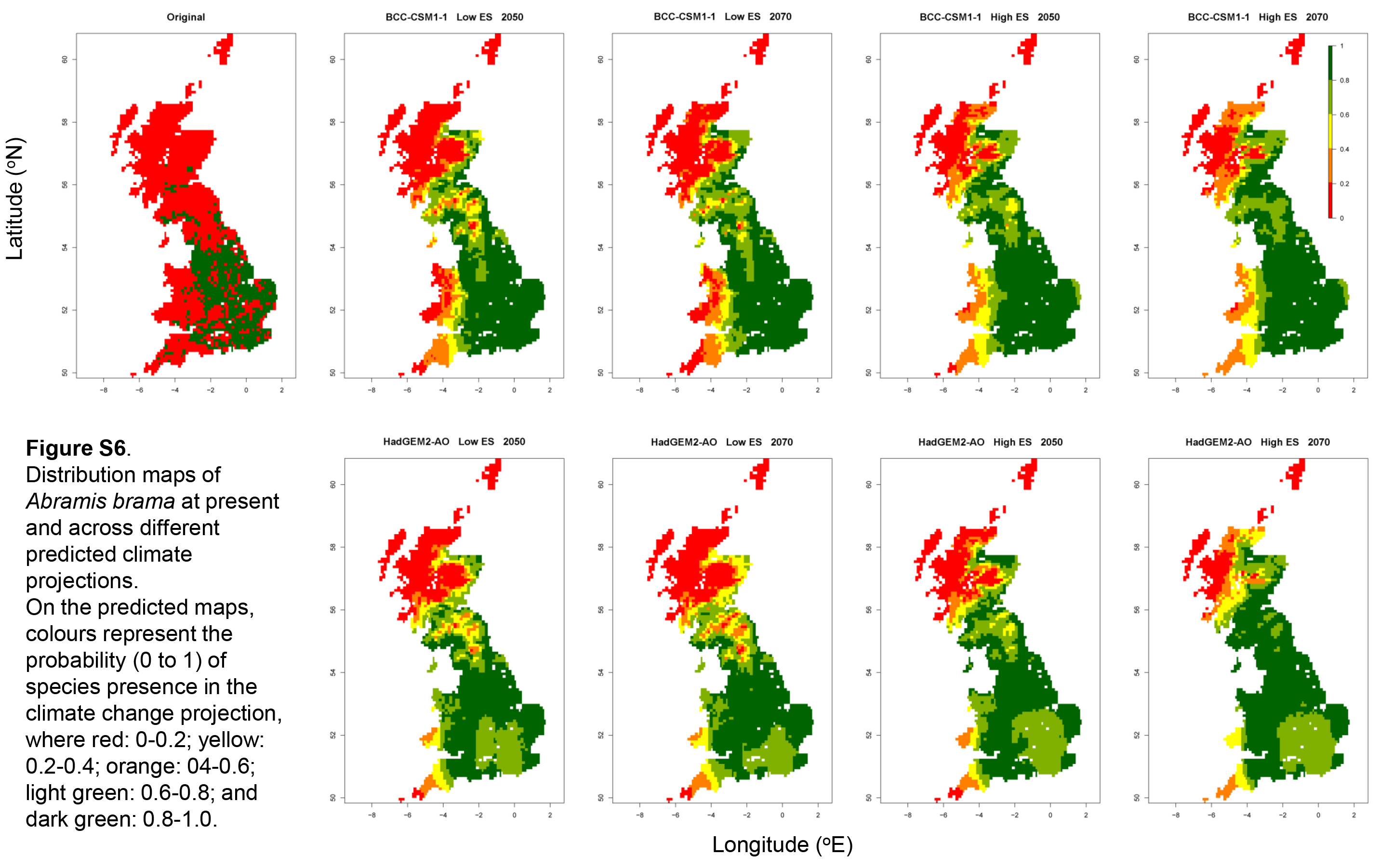

Supplement: Supplementary file 6 — Figure S6. Distribution maps of Abramis brama at present and across different predicted climate projections. [file GCB-22-3221-s006.tif]
